# On the interconnection between the higher-order singular values of real tensors 

**DOI:** 10.1007/s00211-016-0819-9

**Published:** 2016-08-03

**Authors:** Wolfgang Hackbusch, André Uschmajew

**Affiliations:** 1 Max-Planck-Institut Mathematik in den Naturwissenschaften, Inselstraße 22, 04103 Leipzig, Germany; 2 Hausdorff Center for Mathematics & Institute for Numerical Simulation, University of Bonn, 53115 Bonn, Germany

**Keywords:** 15A18, 15A21, 15A69

## Abstract

A higher-order tensor allows several possible matricizations (reshapes into matrices). The simultaneous decay of singular values of such matricizations has crucial implications on the low-rank approximability of the tensor via higher-order singular value decomposition. It is therefore an interesting question which simultaneous properties the singular values of different tensor matricizations actually can have, but it has not received the deserved attention so far. In this paper, preliminary investigations in this direction are conducted. While it is clear that the singular values in different matricizations cannot be prescribed completely independent from each other, numerical experiments suggest that sufficiently small, but otherwise arbitrary perturbations preserve feasibility. An alternating projection heuristic is proposed for constructing tensors with prescribed singular values (assuming their feasibility). Regarding the related problem of characterising sets of tensors having the same singular values in specified matricizations, it is noted that orthogonal equivalence under multilinear matrix multiplication is a sufficient condition for two tensors to have the same singular values in all principal, Tucker-type matricizations, but, in contrast to the matrix case, not necessary. An explicit example of this phenomenon is given.

## Introduction and problem statement

A space ${\mathbb {R}}^{n_{1}}\otimes \dots \otimes {\mathbb {R}}^{n_{d}}$ of higher-order tensors is isomorphic to many different matrix spaces of the form $( \bigotimes _{j\in t}{\mathbb {R}}^{n_{j}}) \otimes (\bigotimes _{i\notin t}{\mathbb {R}}^{n_{i}})$ where $t\subsetneq \{1,\dots ,d\}$, $|t|\ge 1$. Concretely, when identifying tensors with *d*-dimensional arrays of coordinates with respect to an orthonormal tensor product basis, such an isomorphism is realized by reshaping the array into a matrix. The directions in *t* indicate the multi-indices for the rows of the resulting matrix, while the other directions are used for the columns. All these different *matricizations* (also called *unfoldings* or *reshapes* in the literature) of the tensor carry some spectral information in form of their singular value decompositions.

For subsets of *t* that are part of a dimension partition tree, the column spaces of the corresponding matricizations satisfy certain nestedness properties that form the basis for important subspace based low-rank tensor decompositions like the Tucker format [22], the hierarchical Tucker (HT) format  [7, 9], or the tensor train (TT) format [18, 19]. As a by-product, the ranks $r_t$ of the corresponding matricizations, that is, the number of nonzero singular values, are estimated as $r_t \le r_{t_1} \cdots r_{t_s}$, where $t = t_1 \cup \dots \cup t_s$ is a disjoint partition. In contrast, the interconnections between the singular values themselves have not been studied so far.

At first sight, the singular values of different matricizations could be considered as unnatural or artificial characteristics for tensors, as they ignore their multilinear nature. However, as it turns out, they provide crucial measures for the approximability of tensors in the aforementioned low-rank subspace formats. In the pioneering work [3] the higher-order singular value decomposition has been defined, and it has been shown how it can be practically used to obtain quasi-optimal low-rank approximations in the Tucker format with full error control. The approximation is obtained by an orthogonal projection on the tensor product of subspaces spanned by the dominating singular vectors of the corresponding matricizations in ${\mathbb {R}}^{n_{j}}\otimes ( \bigotimes _{i\ne j}{ \mathbb {R}}^{n_{i}})$ (i.e. corresponding to the choices $t=\{j\}$ for $j=1,\dots ,d$). An upper bound of the squared error is then given by the sum of squares of all deleted singular values in all directions. Later, variants of such truncation procedures have been obtained for the TT format  [16, 17] and the HT format [7] with similar error bounds, but involving singular values of some other matricizations of the tensor.

Building on these available, quasi-optimal bounds for low-rank approximations via higher-order versions of SVD truncation, it is understandable that quite some theorems have been stated making simultaneous assumptions on the singular values of certain matricizations of a tensor. This concerns stability of low-rank ODE integrators [12, 14], local convergence of optimization algorithms [20], or approximability by low-rank tensor formats [1], to name a few. Assumed properties of interest are decay rate of and gaps between the singular values, for instance. A principal task would then be to give alternative descriptions of classes of tensors satisfying such assumptions to prevent tautological results or, in worst case, void statements. But this task has turned out to be notoriously difficult. For tensors arising from function discretization, some qualitative statements about the decay of singular values can be obtained from their regularity using explicit analytic approximation techniques by tensor products of (trigonometric) polynomials or wavelets, exponential sum, or cross approximation; see [8, 21] and references therein. But these qualitative implications on the decay of singular values obtained from explicit separable approximations can rarely be made quantitatively precise, for instance, if they contain unknown constants, and also provide little insight on the actual interconnection between different matricizations.

In its purest form the question we are interested in is very simple to state. Given prescribed singular values for some matricizations (having, e.g., some favourable properties), does there exist at all a tensor having these singular values? For a matrix this is of course very easy to answer by simply constructing a diagonal matrix. For tensors it turns out to be quite difficult, and seems to depend on how many matricizations are simultaneously considered.

In the present paper we study this and related questions for the singular values related to the classical higher-order SVD, that is, the singular values of the principal, Tucker-type matricizations that separate single indices $t=\{j\}$. We call the collection of the *d* corresponding singular value vectors the * higher-order singular values*, see Definition 1.1 below. Compared to other systems of matricizations, this framework is the historically most important. It also appears to be the simplest, partly because it is, at least to some extent, very conveniently possible to manipulate the principal matricizations simultaneously via multilinear matrix multiplications. Yet, even in this case, the obtained results remain fragmentary and far from complete. Nevertheless, we consider them as valuable first steps toward future investigations of this important and fascinating subject. Our contributions are as follows.

- We show that not all configurations of higher-order singular values can be feasible. The proof is nonconstructive (Sect. 3.1).
- However, conducted numerical experiments suggest that the singular values for different matricizations are, except for degenerate situations, *locally* independent from each other. That is, in the neighbourhood of a tensor it is possible to slightly perturb, say, only the singular values of the first matricization, while maintaining the singular values of the other ones. This is fundamentally different from the matrix case, since the singular values of a matrix are always the same as the ones of its transpose. However, currently this remains an unproved conjecture Sect. 3.2).
- We propose the method of alternating projections as a heuristic to construct (approximately) tensors with prescribed singular values in certain matricizations (Sect. 3.3).
- The higher-order SVD (HOSVD) is a generalization of the SVD from matrices to tensors. The role of the diagonal matrix of singular values is replaced by the core tensor in the HOSVD, representing the normal form under orthogonal equivalence, and characterized by slice-wise orthogonality properties. We show manifold properties of the set of these core tensors (called HOSVD tensors) in the case of strictly decreasing and positive higher-order singular values (Sect. 2.2).
- We provide an example of two $2\times 2\times 2$ tensors having the same singular values in all three principal matricizations without being orthogonally equivalent (Sect. 2.4).

In this paper we consider real tensors for convenience. Although most concepts seem to generalize to the complex case, some care would be required, e.g., when switching from smooth manifolds to analytic ones.

The rest of this section is devoted to the precise statements of the considered problems. They require some amount of definitions and notation, which will be introduced first.

### Preliminaries, definitions, notation

Let $d\ge 3$ and $n_{1},\dots ,n_{d}\in \mathbb {N}$. We consider the *d*-fold tensor product space ${\mathbb {R}}^{n_{1}}\otimes \dots \otimes {\mathbb {R}} ^{n_{d}}$ as isomorphic to the space ${\mathbb {R}}^{n_{1}\times \dots \times n_{d}}$ of real $n_{1}\times \dots \times n_{d}$ arrays (sometime called *hyper-matrices*). The entries $X_{i_{1},\dots ,i_{d}}$ of ${\mathbf {X}}\in {\mathbb {R}} ^{n_{1}\times \dots \times n_{d}}$ will be indexed by multi-indices $(i_{1},\dots ,i_{d})$, with every $i_{j}$ taking values between 1 and $n_{j}$. For convenience, it will be assumed

$$\begin{aligned} n_{1}\ge n_{2}\ge \dots \ge n_{d} \end{aligned}$$

throughout the paper. Furthermore, we set

$$\begin{aligned} n_{j}^{c}=\prod _{i\ne j}n_{i}. \end{aligned}$$

A tensor ${\mathbf {X}}\in {\mathbb {R}}^{n_{1}\times \dots \times n_{d}}$ admits *d* *principal matricizations* [10, 11]

$$\begin{aligned} M_{{\mathbf {X}}}^{(j)}\in {\mathbb {R}}^{n_{j}\times n_{j}^{c}} \end{aligned}$$

in which the $$i_{j}$$-th row contains all entries $$X_{i_{1},\dots ,i_{d}}$$ with fixed $$i_{j}$$, arranged in some fixed ordering with respect to the remaining multi-indices. The choice of that ordering is not important for our purposes. The matricizations realize the isomorphism between the tensor space $${\mathbb {R}}^{n_{1}}\otimes \dots \otimes {\mathbb {R}}^{n_{d}}$$ and the matrix spaces $${\mathbb {R}}^{n_{j}\times n_{j}^{c}}$$. Note that for tensors $${\mathbf {X}}$$ of order $$d=2 $$ (that is, matrices), the matricizations are given by $$M_{{\mathbf {X}}}^{(1)}={\mathbf {X}}$$ and $$M_{{ \mathbf {X}}}^{(2)}={\mathbf {X}}^{{\mathsf {T}}}$$ (up to permutation).

It will further be convenient to have a notation for the Gram matrix of $M_{{ \mathbf {X}}}^{(j)}$, which will be

$$\begin{aligned} G_{{\mathbf {X}}}^{(j)} = M_{{\mathbf {X}}}^{(j)} \left( M_{{\mathbf {X}} }^{(j)} \right) ^{\mathsf {T}}. \end{aligned}$$

By ${\mathrm {O}}(n)$ we denote the group of real orthogonal $n\times n$ matrices, by $I_n$ the $n \times n$ identity matrix. Each of the matrices $M_{{\mathbf {X}}}^{(j)}$ admits a singular value decomposition

1.1 $$\begin{aligned} M_{{\mathbf {X}}}^{(j)} = U^{(j)}_{\mathbf {X}} \Sigma ^{(j)}_{\mathbf {X}} \left( V^{(j)}_{\mathbf {X}}\right) ^{\mathsf {T}}, \end{aligned}$$

where $U^{(j)}_{\mathbf {X}} \in {\mathrm {O}}(n_{j})$, $(V^{(j)}_{\mathbf {X}})^{\mathsf {T}} V^{(j)}_{\mathbf {X}} = I_{n_j}$, and $\Sigma ^{(j)}_{\mathbf {X}}$ is a diagonal matrix containing the *mode-j singular values* $\sigma _{1}^{(j)} \ge \sigma _{2}^{(j)} \ge \dots \sigma _{n_{j}}^{(j)} \ge 0$ as diagonal elements. We denote $\Lambda ^{(j)}_{\mathbf {X}} = (\Sigma ^{(j)}_{ \mathbf {X}})^{2}$. The diagonal entries of $\Lambda ^{(j)}_{\mathbf {X}}$ are the ordered eigenvalues of $G_{{\mathbf {X}}}^{(j)}$.

#### Definition 1.1

Let $${\mathbf {X}} \in {\mathbb {R}}^{n_{1} \times \dots \times n_{d} }$$.

1. For $j=1,\dots ,d$, the vector
  $$\begin{aligned} {{\varvec{\sigma }}}^{(j)}_{\mathbf {X}} = {{\mathrm{{diag}}}}\left( \Sigma ^{(j)}_{\mathbf {X}}\right) = \left( \sigma ^{(j)}_{1},\dots ,\sigma ^{(j)}_{n_{j}}\right) \in {\mathbb {R}}^{n_{j}}_{+} \end{aligned}$$
  is called the *vector of mode-j singular values*. The tuple
  $$\begin{aligned} {\varvec{\Sigma }}_{\mathbf {X}} = \left( {\varvec{\sigma }}^{(1)}_{\mathbf {X} },\dots ,{\varvec{\sigma }}^{(d)}_{\mathbf {X}}\right) \in {\mathbb {R}}^{n_{1}}_{+} \times \dots \times {\mathbb {R}}^{n_{d}}_{+} \end{aligned}$$
  is called the set of *higher-order singular values* of the tensor ${\mathbf {X}}$.
2. Correspondingly, for $j=1,\dots ,d$, the vector
  $$\begin{aligned} {{\varvec{\lambda }}}^{(j)}_{\mathbf {X}} = {{\mathrm{{diag}}}}\left( \Lambda ^{(j)}_{\mathbf {X} }\right) = \left( \left( \sigma ^{(j)}_{1}\right) ^{2},\dots ,\left( \sigma ^{(j)}_{n_{j}}\right) ^{2}\right) \in {\mathbb {R} }^{n_{j}}_{+} \end{aligned}$$
  is called the *vector of mode-j Gramian eigenvalues*. The tuple
  $$\begin{aligned} {\varvec{\Lambda }}_{\mathbf {X}} = \left( {\varvec{\lambda }}^{(1)} _{\mathbf {X}},\dots ,{\varvec{\lambda }}^{(d)}_{\mathbf {X}}\right) \end{aligned}$$
  is called the set of *higher-order Gramian eigenvalues* of the tensor ${\mathbf {X}}$.
3. The *multilinear rank* of the tensor ${\mathbf {X}}$ is the tuple ${\mathbf {r} }_{\mathbf {X}} = (r^{(1)},\dots ,r^{(d)})$ with $r^{(j)} = {{\mathrm{{rank}}}}( M_{{\mathbf {X}}}^{(j)} ) = {{\mathrm{{rank}}}}( G_{{\mathbf {X}}}^{(j)} )$ being equal to the number of nonzero entries of ${\varvec{\sigma }}^{(j)}_{\mathbf {X}}$.
4. The tensor ${\mathbf {X}}$ is called *non-singular*, if ${\mathbf {r}}_{\mathbf {X}} = (n_{1},\dots ,n_{d})$.

We note that for matrices the definition of ‘non-singular’ coincides with the usual definition (in particular, it enforces $n_{1} = n_{2}$). In general, the following is true.

#### Proposition 1.2

There exists a non-singular tensor in $\mathbb {R}^{n_1 \times \dots \times n_d}$ if and only if the following *compatibility conditions* hold:

1.2 $$\begin{aligned} n_{j} \le n_{j}^{c}, \quad j=1,2,\dots ,d. \end{aligned}$$

In this case the set of non-singular tensors is open and dense in ${\mathbb {R}}^{n_{1} \times \dots \times n_{d}}$.

#### Proof

Consider *j* fixed. By isomorphy and known results on matrices, it is clear that the set of all ${\mathbf {X}}$ with $M_{{\mathbf {X}}}^{(j)}$ being of rank $n_j$ is not empty, open, and dense if and only if $n_j \le n_j^c$. The set of non-singular tensors is the intersection of these sets for $j=1,\dots ,d$. As such, it is also open and dense. $\square$

Let $\Vert \cdot \Vert _F$ denote the Frobenius norm of matrices and tensors, and $\Vert \cdot \Vert _2$ the standard Euclidean norm for vectors. Since matricization of a tensor is an isometric isomorphism in Frobenius norm, and since it holds $\Vert M_{{\mathbf {X}}}^{(j)}\Vert _{F}=\Vert {\varvec{ \sigma }}_{{\mathbf {X}}}^{(j)}\Vert _{2}$, an obvious observation for higher-order singular values is

$$\begin{aligned} {\Vert {\varvec{\sigma }}_{{\mathbf {X}}}^{(1)}\Vert }_{2}=\dots ={\Vert { \varvec{\sigma }}_{{\mathbf {X}}}^{(d)}\Vert }_{2}=\Vert {\mathbf {X}} \Vert _{F}^{{}}. \end{aligned}$$

Therefore, we can focus in the following on tensors ${\mathbf {X}}$ on the unit sphere

$$\begin{aligned} {\mathscr {S}}=\left\{ {\mathbf {X}}\in {\mathbb {R}}^{n_{1}\times \dots \times n_{d}} :\Vert {\mathbf {X}}\Vert _{F}=1\right\} , \end{aligned}$$

and hence higher-order singular values ${\varvec{\Sigma }}$ in the set

$$\begin{aligned} {\mathfrak {S}}_{\ge }={\mathfrak {S}_{\ge }(n_1,\dots ,n_d)}={S} _{\ge }^{(1)}\times \dots \times {S}_{\ge }^{(d)}, \end{aligned}$$

where ${S}_{\ge }^{(j)}$ denotes the set of all nonnegative, decreasingly ordered vectors on the Euclidean unit sphere in ${\mathbb {R}} ^{n_{j}}$. For most results, however, it will be necessary to further restrict to the set $\mathscr {S}^{*}$ of non-singular tensors having strictly decreasing mode-*j* singular values in every direction *j*. Therefore, we also introduce the notation

$$\begin{aligned} {\mathfrak {S}}_{>}={\mathfrak {S}_{>}(n_1,\dots ,n_d)} ={S} _{>}^{(1)}\times \dots \times {S}_{>}^{(d)}\subset {\mathfrak {S}}_{\ge }, \end{aligned}$$

where each $S_{>}^{(j)}$ contains the unit norm vectors in $\mathbb {R}^{n_j}$ with strictly decreasing *and* strictly positive entries. Then

$$\begin{aligned} {\mathscr {S}}^{*}=\{{\mathbf {X}}\in {\mathscr {S}}:{\varvec{ \Sigma }}_{{\mathbf {X}}}\in {\mathfrak {S}}_{>}\}. \end{aligned}$$

Note that we do not introduce a notation for the slightly larger set of all non-singular tensors in ${\mathscr {S}}$. The main technical advantage of tensors in $\mathscr {S}^{*}$ is that all principal unfoldings admit essentially unique singular value decompositions.

The following two facts are useful to know, and follow immediately from the matrix case.

#### Proposition 1.3

The function ${\mathbf {X}} \mapsto {\varvec{\Sigma }}_{\mathbf {X}}$ is continuous on ${\mathscr {S}}$. Assuming (1.2), the set ${\mathscr {S} }^{*}$ is relatively open and dense in ${\mathscr {S}}$.

#### Proof

The continuity of ${\varvec{\Sigma }}_{\mathbf {X}}$ as a function of ${\mathbf {X}}$ follows by isomorphy to ${\mathbb {R}}^{n_j \times n_j^c}$ from the continuity of each ${\varvec{\sigma }}^{(j)}_{\mathbf {X}}$ as a function of $M_{{\mathbf {X}}}^{(j)}$. The proof that ${\mathscr {S}}^{*}$ is relatively open and dense in ${\mathscr {S}}$ is analogous to the proof of Proposition 1.2. $\square$

### Problem statement

Regarding the higher-order singular values of tensors a principle question of interest is the following one.

#### Problem 1.4

(*Feasible higher-order singular values*) Given ${\varvec{\Sigma }} \in {\mathfrak {S}}_{\ge }$, does there exist a tensor ${\mathbf {X}} \in {\mathscr {S}}$ such that ${\varvec{\Sigma }}_{\mathbf {X}} = {\varvec{\Sigma }}$?

Such ${\varvec{\Sigma }}$ will be called a *feasible*. We define

$$\begin{aligned} {\mathfrak {F}} = {\mathfrak {F}} (n_{1},\dots ,n_{d}) = \{ {\varvec{\Sigma }} \in {\mathfrak {S}}_{\ge } :{\varvec{\Sigma }}\, \text {is feasible}\}. \end{aligned}$$

In this generality, Problem 1.4 appears to be quite hard, and will not be satisfactorily solved in this article. At least, as a first result, we are able to show that not all ${\varvec{\Sigma }} \in {\mathfrak {S}}_{\ge }$ are feasible: ${\mathfrak {F}} \ne \mathfrak {S}_\ge$. The argument, however, is non-constructive, see Sect. 3.1.

A relaxed question of a more qualitative nature is the following one.

#### Problem 1.5

(*Properties of* ${\mathfrak {F}}$) What are the topological properties of the set ${\mathfrak {F}}$ as a subset of ${\mathfrak {S}}_{\ge }$? Does it, for instance, have positive (relative) Lebesgue measure?

Numerical experiments with random tensors seem to indicate that the answer to the second question could be positive when $d\ge 3$, but we are not able to prove it. So it remains a conjecture. In fact, we conjecture that for every $\mathbf {X}$ in $\mathscr {S^{*}}$ it holds that $\varvec{\Sigma }_{\mathbf {X}}$ is an interior point of $\mathfrak {F}$, see Sect. 3.2. A striking implication of this conjecture is that given ${\mathbf {X}} \in \mathscr {S}^{*}$, its high-order singular values in different directions can be perturbed independently from each other without loosing feasibility (local independence of high-order singular values). In Sect. 3.3 we will present a heuristic approach to do this using an alternating projection method, which seems to work quite reliably for small perturbations, although we are currently neither able to prove its convergence nor that limit points must have the desired property.

To approach Problems 1.4 and 1.5, it seems useful to also study the following problem, which is of some interest in itself.

#### Problem 1.6

(*Tensors with same higher-order singular values*) Given ${\mathbf {X}} \in {\mathscr {S}}$, characterize sets of tensors having the same singular values ${\varvec{\Sigma }}_{\mathbf {X}}$ as ${\mathbf {X}}$.

The corresponding equivalence classes for tensors in ${\mathscr {S}}$ and ${ \mathscr {S}}^{*}$ are denoted by

$$\begin{aligned} {\mathscr {S}}_{\mathbf {X}} = \{ {\mathbf {Y}} \in {\mathscr {S}} :{\varvec{\Sigma }}_{\mathbf {Y}} = {\varvec{\Sigma }}_{\mathbf {X}} \}, \quad {\mathscr {S}}_{\mathbf {X}}^{*} = \{ {\mathbf {Y}} \in {\mathscr {S}}^{*} :{\varvec{\Sigma }}_{\mathbf {Y}} = {\varvec{\Sigma }}_{ \mathbf {X}} \}. \end{aligned}$$

The next Sect. 2 provides some results related to Problem 1.6. It is observed that orbits of orthogonally equivalent tensors provide trivial examples of subsets of tensors having the same higher-order singular values. However, other than in the matrix case, their dimension is too small to provide a complete description. Via the tool of HOSVD tensors, which serve as normal forms in the orbits of orthogonally equivalent tensors, we are able to construct an example of two tensors with the same higher-order singular values that are not orthogonally equivalent.

## Tensors with the same higher-order singular values

In this section we focus on equivalence classes of tensors having the same higher-order singular values.

### Orthogonally equivalent tensors

We recall a fact from matrices: Two rectangular matrices $X, Y \in {\mathbb {R}}^{n \times n^{\prime }}$, $n \le n^{\prime }$, have the same singular values, if and only if they are orthogonally equivalent, that is, if there exists $U \in {\mathrm {O}}(n)$ and $V \in {\mathrm {O}}(n^{\prime })$ such that

$$\begin{aligned} U X V^{\mathsf {T}} = Y. \end{aligned}$$

This definition of orthogonal equivalence can be generalized to tensors using the multilinear matrix multiplication, see, e.g., [5, 13]. We consider the product unitary group

$$\begin{aligned} {\mathrm {O}}(n_{1}\times \dots \times n_{d})={\mathrm {O}}(n_{1})\times { \mathrm {O}}(n_{2})\times \dots \times {\mathrm {O}}(n_{d}). \end{aligned}$$

The left action $(U^{(1)},\dots ,U^{(d)})\cdot {\mathbf {X}}$ of this group on ${\mathbb {R}}^{n_{1}\times \dots \times n_{d}}$ is defined as the canonical action of the tensor product operator $U^{(1)}\otimes \dots \otimes U^{(d)}$ on ${\mathbb {R}}^{n_{1}}\otimes \dots \otimes {\mathbb {R}}^{n_{d}}$ in the sense that

$$\begin{aligned} (U^{(1)},\dots ,U^{(d)})\cdot \left( \sum _{k=1}^{r}x_{k}^{(1)}\otimes \dots \otimes x_{k}^{(d)}\right) =\sum _{k=1}^{r}U^{(1)}x_{k}^{(1)}\otimes \dots \otimes U^{(d)}x_{k}^{(d)}. \end{aligned}$$

In terms of matricizations, in a slight abuse of notation, we note that

2.1 $$\begin{aligned} M_{(U^{(1)},\dots ,U^{(d)})\cdot {\mathbf {X}}}^{(j)}=U^{(j)}M_{{\mathbf {X}} }^{(j)}\left( U^{(1)}\otimes \dots \otimes U^{(j-1)}\otimes U^{(j+1)}\otimes \dots \otimes U^{(d)}\right) ^{{\mathsf {T}}}, \end{aligned}$$

cf. [8, Lemma 5.6]. In particular, since $U^{(1)} \otimes \dots \otimes U^{(j-1)} \otimes U^{(j+1)} \otimes \dots \otimes U^{(d)} \in {\mathrm {O}}(n_{j}^{c})$, it holds

2.2 $$\begin{aligned} G_{(U^{(1)},\dots ,U^{(d)}) \cdot {\mathbf {X}}}^{(j)} = U^{(j)} G_{{\mathbf {X}} }^{(j)} (U^{(j)})^{\mathsf {T}} \end{aligned}$$

for $j=1,\dots ,d$. For matrices ($d=2$), these formulas define orthogonal equivalence, which motivates the following generalization.

#### Definition 2.1

(*see* [5]) Two tensors ${\mathbf {X}},{\mathbf {Y}} \in {\mathbb {R}}^{n_{1} \times \dots \times n_{d}}$ are called *orthogonally equivalent*, if there exists $(U^{(1)},\dots ,U^{(d)}) \in {\mathrm {O}}(n_{1} \times \dots \times n_{d})$ such ${\mathbf {Y}} {=} (U^{(1)},\dots ,U^{(d)})~\cdot {\mathbf {X}}$.

From (2.2), we draw a trivial but important conclusion.

#### Proposition 2.2

If two tensors are orthogonally equivalent, then they have the same higher-order singular values.

In particular, the *orbit* of each ${\mathbf {X}}$ under the group action contains only tensors with identical higher-order singular values.

#### Proposition 2.3

Let ${\mathbf {X}}\in {\mathscr {S}} ^{*}$. Then the *orbit* ${\mathrm {O}}(n_{1}\times \dots \times n_{d})\cdot {\mathbf {X}}$ is a locally smoothly embedded submanifold of ${\mathscr {S}}^{*}$ of dimension

$$\begin{aligned} \dim ({\mathrm {O}}(n_{1}\times \dots \times n_{d})\cdot {\mathbf {X}} )=\dim ({\mathrm {O}}(n_{1}\times \dots \times n_{d}))=\sum _{j=1}^{d}\frac{1}{2}n_{j}(n_{j}-1). \end{aligned}$$

#### Proof

We write ${\mathrm {O}}$ instead of ${\mathrm {O}}(n_1 \times \dots \times n_d)$. Consider the canonical map $\theta _{\mathbf {X}}:{\mathrm {O}}\rightarrow {\mathscr {S}}^{*}$, $(U^{(1)},\dots ,U^{(d)}) \mapsto (U^{(1)},\dots ,U^{(d)}) \cdot {\mathbf {X}}$, whose image is ${\mathrm {O}}\cdot {\mathbf {X}}$. Since $\theta _{\mathbf {X}}$ is of constant rank [6, §16.10.2] and easily shown to be locally injective (uniqueness of left singular vectors up to sign flipping for ${\mathbf {X}}\in {\mathscr {S}}^{*}$), it is already an immersion [6, §16.8.8.(iv)]. The result is now standard, see, e.g., [6, §16.8.8.(ii)]. $\square$

For ${\mathbf {X}} \in {\mathscr {S}} \setminus {\mathscr {S}}^{*}$ the dimension of ${\mathrm {O}}(n_{1} \times \dots \times n_{d}) \cdot {\mathbf {X}}$ can be smaller than $\dim {\mathrm {O}}(n_{1} \times \dots \times n_{d})$. Note that we did not attempt to prove or disprove that the orbits are globally embedded submanifolds.

### HOSVD tensors

The compact Lie group ${\mathrm {O}}(n_{1} \times \dots \times n_{d})$ acts freely on ${\mathscr {S}}^{*}$. It also acts properly (since it is compact and acts continuously). By a general theorem (e.g. [6, § 16.10.3]), the quotient manifold ${\mathscr {S}}^{*} / {\mathrm {O}} (n_{1} \times \dots \times n_{d})$ of equivalence classes exists, and the canonical mapping ${ \mathscr {S}}^{*} \rightarrow {\mathscr {S}}^{*} / {\mathrm {O}}(n_{1} \times \dots \times n_{d})$ is a submersion. A concrete realization of this abstract quotient manifold is the set ${\mathscr {H}}^{*}$ of regular HOSVD tensors which is now introduced.

Given ${\mathbf {Y}}$, let $U^{(j)}_{\mathbf {Y}}$ denote a matrix of left singular vectors of ${{M}}^{(j)}_{\mathbf {Y}}$ as in  (1.1). By (2.1), ${ \mathbf {X}} = ((U_{\mathbf {Y}}^{(1)})^{\mathsf {T}},\dots ,(U_{\mathbf {Y} }^{(d)})^{\mathsf {T}}) \cdot {\mathbf {Y}}$ has the matricizations

$$\begin{aligned} M_{{\mathbf {X}}}^{(j)} = \Sigma ^{(j)}_{\mathbf {Y}}\left( V_{\mathbf {Y}}^{(j)}\right) ^{\mathsf {T}}\left( U^{(1)}_{\mathbf {Y}} \otimes \dots \otimes U^{(j-1)}_{\mathbf {Y}} \otimes U^{(j+1)}_{\mathbf {Y}} \otimes \dots \otimes U^{(d)}_{\mathbf {Y}}\right) . \end{aligned}$$

In particular, the rows of $(V_{\mathbf {Y}}^{(j)})^{\mathsf {T}}(U^{(1)}_{ \mathbf {Y}} \otimes \dots \otimes U^{(j-1)}_{\mathbf {Y}} \otimes U^{(j+1)}_{ \mathbf {Y}} \otimes \dots \otimes U^{(d)}_{\mathbf {Y}})$ are right singular vectors of $M_{{\mathbf {X}}}^{(j)}$, the left singular vectors are unit vectors, and the singular values are the same as of ${ \mathbf {Y}}$, that is, $\Sigma ^{(j)}_{\mathbf {X}} = \Sigma ^{(j)}_{\mathbf {Y}}$. Hence ${\mathbf {X}}$ has the specific property that

2.3 $$\begin{aligned} G_{{\mathbf {X}}}^{(j)} = M_{{\mathbf {X}}}^{(j)} \left( M_{{\mathbf {X}}}^{(j)} \right) ^{ \mathsf {T}} = \left( \Sigma ^{(j)}_{\mathbf {X}}\right) ^{2} = \Lambda ^{(j)}_{\mathbf {X}}, \quad j=1,\dots ,d, \end{aligned}$$

is a diagonal matrix of decreasing eigenvalues. The reverse relation

$$\begin{aligned} {\mathbf {Y}} = \left( U^{(1)}_{\mathbf {Y}}, \dots , U^{(d)}_{\mathbf {Y}}\right) \cdot { \mathbf {X}} \end{aligned}$$

between ${\mathbf {X}}$ and ${\mathbf {Y}}$ is called the *higher-order singular value decomposition (HOSVD)* of ${\mathbf {Y}}$ and has been introduced by De Lathauwer et al. [3].

#### Definition 2.4

Tensors satisfying (2.3) are called *HOSVD tensors*. The subset of HOSVD tensors in ${\mathscr {S}}$ is denoted by ${\mathscr {H}}$, and the subset of HOSVD tensors in $\mathscr {S}^{*}$ by ${\mathscr {H}}^{*} = {\mathscr {H}} \cap {\mathscr {S}}^{*}$.

HOSVD tensors can be regarded as representatives of orbits ${\mathrm {O}} (n_{1} \times \dots \times n_{d}) \cdot {\mathbf {X}}$ of orthogonally equivalent tensors. For ${\mathbf {X}} \in {\mathscr {S}}^{*}$, the representatives are essentially unique as stated next. Here it is instructive to note that for square matrices, the set $\mathscr {H}^{*}$ consists of regular diagonal matrices with strictly decreasing diagonal entries.

#### Proposition 2.5

Let ${\mathbf {X}}, {\mathbf {Y}} \in {\mathscr {H}}^{*}$ be two HOSVD tensors. If ${\mathbf {X}}$ and ${\mathbf {Y}}$ are orthogonally equivalent, that is, ${\mathbf {Y}} = (U^{(1)},\dots ,U^{(d)}) \cdot {\mathbf {X}}$, then the $U^{(j)}$ must be diagonal orthogonal matrices (i.e. with values $\pm 1$ on the diagonal).

The proof is immediate from (2.2),  (2.3), and the uniqueness of orthogonal diagonalization up to sign flipping in the case of mutually distinct eigenvalues. Comparing with the explicit form  (2.1), we see that the action of $(U^{(1)}, \dots , U^{(d)}) \cdot {\mathbf {X}}$ with diagonal $U^{(j)}$ with $\pm 1$ entries results in *some* sign flipping pattern for the entries of ${\mathbf {X}}$. This provides the following, sometimes useful necessary condition.

#### Proposition 2.6

If two HOSVD tensors ${\mathbf {X}}, {\mathbf {Y}} \in {\mathscr {H}}^{*}$ are orthogonally equivalent, then $|X_{i_{1},\dots ,i_{d}}|= |Y_{i_{1},\dots ,i_{d}}|$. In particular, ${\mathbf {X}}$ and ${\mathbf {Y}}$ have the same zero pattern.

We now turn to the manifold properties of ${\mathscr {H}}^{*}$.

#### Theorem 2.7

The set ${\mathscr {H}}^{*}$ is a smooth embedded submanifold of ${\mathscr {S} }^{*}$ of dimension

$$\begin{aligned} \dim ({\mathscr {H}}^{*}) = n_{1} \cdots n_{d} - \sum _{j=1}^{d}\frac{1}{2} n_{j}(n_{j} - 1) = \dim ({\mathscr {S}}^{*}) - \dim ({\mathrm {O}}(n_{1} \times \dots \times n_{d})). \end{aligned}$$

#### Proof

The formal setting is as follows. We denote by ${\mathbb {R}}^{n_j \times n_j}_{\text {sym}}$ the space of symmetric $n_j \times n_j$ matrices, by ${\mathbb {R}}^{n_j \times n_j}_{\text {sym},0}$ the subspace with zeros on the diagonal, by $\pi _j(Z) = Z - {{\mathrm{{diag}}}}(Z)$ the orthogonal projection from ${\mathbb {R}}^{n_j \times n_j}_{\text {sym}}$ onto ${\mathbb {R}}^{n_j \times n_j}_{\text {sym},0}$, and $p = \pi _1 \times \dots \times \pi _d$. Consider

2.4 $$\begin{aligned} g :{\mathscr {S}^{*}} \rightarrow {\mathbb {R}}^{n_1 \times n_1}_{\text {sym}} \times \dots \times {\mathbb {R}}^{n_d \times n_d}_{\text {sym}}, \quad {\mathbf {X}}\mapsto ( G_{{\mathbf {X}}}^{(1)} ,\dots , G_{{\mathbf {X}}}^{(d)} ). \end{aligned}$$

Then $f = p \circ g$ is a smooth map from ${\mathscr {S}}^{*}$ to ${\mathbb {R}}^{n_1 \times n_1}_{\text {sym},0} \times \dots \times {\mathbb {R}}^{n_d \times n_d}_{\text {sym},0}$, and, by Definition 2.4, we have

$$\begin{aligned} {\mathscr {H}}^{*} = f^{-1}(0). \end{aligned}$$

Since $\dim ({\mathbb {R}}^{n_1 \times n_1}_{\text {sym},0} \times \dots \times {\mathbb {R}}^{n_d \times n_d}_{\text {sym},0}) =\dim ({\mathrm {O}}(n_1 \times \dots \times n_d)) = \sum _{j=1}^d\frac{1}{2} n_j(n_j - 1),$ the assertion will follow from the regular value theorem, if we show that $f'({\mathbf {X}})$ is surjective for every ${\mathbf {X}}\in \mathscr {H}^{*}$. To prove the latter, we show that the range of $f'({\mathbf {X}})$ contains the spaces ${\mathscr {W}}_j = \{0\} \times \cdots \times \{0\} \times {\mathbb {R}}^{n_j \times n_j}_{\text {sym},0} \times \{0\} \times \dots \times \{0\}$ for $j =1,\dots ,d$. We demonstrate this for $j=1$. Consider the map

$$\begin{aligned} \varphi :{\mathrm {O}}(n_1) \rightarrow {\mathscr {S}}^{*}, \quad U \mapsto (U, I_{n_2}, \dots , I_{n_d}) \cdot {\mathbf {X}}. \end{aligned}$$

For brevity, we set $I = I_{n_1}$. Since $\varphi (I) = {\mathbf {X}}$, it follows from the chain rule that the range of $f'({\mathbf {X}})$ contains the range of $(f \circ \varphi )'(I)$. We show that the latter equals ${\mathscr {W}}_1$. Since ${\mathbf {X}}\in \mathscr {H}^{*}$, we have $(f \circ \varphi )(U) = p(U \Lambda _{\mathbf {X}}^{(1)} U^{\mathsf {T}}, \Lambda _{\mathbf {X}}^{(2)},\dots ,\Lambda _{\mathbf {X}}^{(d)})$. Further noting that the tangent space to ${\mathrm {O}}(n_1)$ at *I* is the space ${\mathbb {R}}^{n_1 \times n_1}_{\text {skew}}$ of skew-symmetric $n_1 \times n_1$ matrices, we see that

$$\begin{aligned} (f \circ \varphi )'(I) :{\mathbb {R}}^{n_1 \times n_1}_{\text {skew}} \rightarrow {\mathscr {W}}_1, \quad H \mapsto p (H \Lambda _{\mathbf {X}}^{(1)} + \Lambda _{\mathbf {X}}^{(1)} H^{\mathsf {T}}, 0,\dots ,0). \end{aligned}$$

As $\dim ({\mathscr {W}}_1) = \frac{1}{2}n_1(n_1 - 1) = \dim ({\mathbb {R}}^{n_1 \times n_1}_{\text {skew}})$, it is enough to show that $(f \circ \varphi )'(I)$ is injective in order to finish the proof. This now follows from the fact that, by definition of ${\mathscr {H}}^{*}$, the diagonal entries of ${\varvec{\Lambda }}_{\mathbf {X}}^{(1)}$ are strictly decreasing, as it implies that $H \Lambda _{\mathbf {X}}^{(1)} + \Lambda _{\mathbf {X}}^{(1)} H^{\mathsf {T}}= H \Lambda _{\mathbf {X}}^{(1)} - \Lambda _{\mathbf {X}}^{(1)} H$ cannot be diagonal for skew-symmetric $H \ne 0$. This, however, is equivalent to injectivity of $(f \circ \varphi )'(I)$ as given above. $\square$

#### Remark 2.8

In our definition (2.3) of HOSVD tensors we required the diagonal elements of $G_{\mathbf {X}}^{(j)}$ to be decreasing. This has advantages and drawbacks. One advantage are the narrower uniqueness properties leading to the practical condition in Proposition 2.6. A disadvantage is that it is more difficult to design HOSVD tensors “by hand” as in Sect. 2.4. Alternatively, one may define a set $\tilde{\mathscr {H}}$ by just requiring the $G_{\mathbf {X}}^{(j)}$ to be diagonal. Then for every ${\mathbf {X}}\in \tilde{\mathscr {H}}$ we have $(P^{(1)},\dots ,P^{(d)}) \cdot {\mathbf {X}}\in \mathscr {H}$, where $P^{(j)}$ are permutation matrices that sort the diagonal entries of $G_{\mathbf {X}}^{(j)}$ accordingly. For mutually distinct eigenvalues the choice of $P^{(j)}$ is unique. The corresponding set $\tilde{\mathscr {H}}^{*}$ is therefore the finite disjoint union of sets $(P^{(1)},\dots ,P^{(d)}) \cdot \mathscr {H}^{*}$ over all $P^{(j)}$, and as such also an embedded submanifold of $\mathscr {S}^{*}$.

### Degrees of freedom

A principal challenge in understanding the interconnection between higher-order singular values of tensors arises from the fact that, in contrast to the matrix case, the converse statement of Proposition 2.2 is in general not true when $d \ge 3$. Tensors may have the same higher-order singular values without being orthogonally equivalent. This can be seen from the following heuristic.

The set ${\mathscr {S}}^{*}$ is open and dense in ${\mathscr {S}}$ by Proposition 1.3, and therefore is a smooth manifold of dimension

$$\begin{aligned} \dim ({\mathscr {S}}^{*})=(n_{1}\dots n_{d})-1. \end{aligned}$$

The set ${\mathfrak {S}}_{>}$ is an open subset of Cartesian products of spheres and hence of dimension

$$\begin{aligned} \dim ({\mathfrak {S}}_{>})=(n_{1}+\dots +n_{d})-d. \end{aligned}$$

Therefore, given ${\mathbf {X}}\in {\mathscr {S}}^{*}$, we expect the set ${ \mathscr {S}}_{\mathbf {X}}^{*}$ of tensors having the same higher-order singular values as ${\mathbf {X}}$ to be at least of “dimension”

$$\begin{aligned} \dim ({\mathscr {S}}^{*})-\dim ({\mathfrak {S}}_{>})=(n_{1}\cdots n_{d})-(n_{1}+\dots +n_{2})+(d-1). \end{aligned}$$

When $d\ge 3$, by Proposition 2.3, this set cannot only consist of tensors that are orthogonally equivalent to ${\mathbf {X}}$.^1^ In fact, for large *d*, the orthogonally equivalent tensors will only be a very “low-dimensional” subset of ${\mathscr {S}}_{\mathbf { X}}^{*}$.

### A non-equivalent example

The previous considerations suggest that there must exist tensors having the same higher-order singular values without being orthogonally equivalent. We construct here an example of size $2 \times 2 \times 2$ using Proposition 2.6. Let us shortly count the degrees of freedom in this situation. The Euclidean unit sphere ${\mathscr {S} }$ is of dimension seven, the set ${\mathfrak {S}}_{\ge }$ of potential tuples of higher-order singular values is of dimension three, while orbits ${ \mathrm {O}}(2 \times 2 \times 2) \cdot {\mathbf {X}}$ of orthogonally equivalent tensors are of dimension at most three, too. This indicates for every ${ \mathbf {X}} \in {\mathscr {S}}$ an at least one-dimensional set of non-equivalent tensor with same higher-order singular values.

Using a common slice-wise notation of tensors, we consider (currently not normalized)

$$\begin{aligned} {\mathbf {X}}=\left[ \begin{array}{ll} -2 &{}\quad 1 \\ 1 &{}\quad 1 \end{array} \right| \left. \begin{array}{ll} -1 &{}\quad -1 \\ -1 &{}\quad 0 \end{array} \right] . \end{aligned}$$

The three matricizations are $$M_{{\mathbf {X}}}^{(1)}= \begin{bmatrix} -2&\quad 1&\quad -1&\quad -1\\ 1&\quad 1&\quad -1&\quad 0 \end{bmatrix}$$, $$M_{{\mathbf {X}}}^{(2)}= \begin{bmatrix} -2&\quad 1&\quad -1&\quad -1\\ 1&\quad 1&\quad -1&\quad 0 \end{bmatrix}$$, and $$M_{{\mathbf {X}}}^{(3)}= \begin{bmatrix} -2&\quad 1&\quad 1&\quad 1\\ -1&\quad -1&\quad -1&\quad 0 \end{bmatrix}$$. In all three matricizations the rows are orthogonal, and the norm of the first row is larger than the norm of the second one. This shows that ${\mathbf {X}}$ is a HOSVD tensor. Its squared higher-order singular values are

$$\begin{aligned} {\varvec{\Lambda }}_{\mathbf {X}}=\left[ {\varvec{\lambda }}_{\mathbf {X} }^{(1)},{\varvec{\lambda }}_{\mathbf {X}}^{(2)},{\varvec{\lambda }}_{ \mathbf {X}}^{(3)}\right] =\left[ \left( {\begin{array}{c}7\\ 3\end{array}}\right) ,\left( {\begin{array}{c}7\\ 3\end{array}}\right) ,\left( {\begin{array}{c}7\\ 3\end{array}}\right) \right] . \end{aligned}$$

In particular, ${\mathbf {X}}/\Vert {\mathbf {X}}\Vert _{F}\in {\mathscr {S}} ^{*}$. As a second tensor consider

$$\begin{aligned} {\mathbf {Y}}=\left[ \begin{array}{ll} 3/\sqrt{2} &{}\quad -1 \\ -1 &{}\quad -1/\sqrt{2} \end{array} \right| \left. \begin{array}{ll} 1 &{}\quad 1/\sqrt{2} \\ 1/\sqrt{2} &{}\quad 1 \end{array} \right] . \end{aligned}$$

One checks again that all three matricizations $$M_{{\mathbf {Y}}}^{(1)}= \begin{bmatrix} 3/\sqrt{2}&\quad -1&\quad 1&\quad 1/\sqrt{2}\\ -1&\quad -1/\sqrt{2}&\quad 1/\sqrt{2}&\quad 1 \end{bmatrix}$$, $$M_{{\mathbf {Y}}}^{(2)}= \begin{bmatrix} 3/\sqrt{2}&\quad -1&\quad 1&\quad 1/\sqrt{2}\\ -1&\quad -1/\sqrt{2}&\quad 1/\sqrt{2}&\quad 1 \end{bmatrix}$$, and $$M_{{\mathbf {Y}}}^{(3)}= \begin{bmatrix} 3/\sqrt{2}&\quad -1&\quad -1&\quad -1 / \sqrt{2}\\ 1&\quad 1/\sqrt{2}&\quad 1/\sqrt{2}&\quad 1 \end{bmatrix}$$ have orthogonal rows with squared row norms

$$\begin{aligned} {\varvec{\Lambda }}_{\mathbf {Y}}=\left[ {\varvec{\lambda }}_{\mathbf {Y} }^{(1)},{\varvec{\lambda }}_{\mathbf {Y}}^{(2)},{\varvec{\lambda }}_{ \mathbf {Y}}^{(3)}\right] =\left[ \left( {\begin{array}{c}7\\ 3\end{array}}\right) ,\left( {\begin{array}{c}7\\ 3\end{array}}\right) ,\left( {\begin{array}{c}7\\ 3\end{array}}\right) \right] . \end{aligned}$$

This shows that ${\mathbf {X}}/\Vert {\mathbf {X}}\Vert _{F}$ and ${\mathbf {Y}} /\Vert {\mathbf {Y}}\Vert _{F}$ are two HOSVD tensors in ${\mathscr {S}}^{*}$ with the same set of higher-order singular values. By Proposition 2.6, they are not orthogonally equivalent.

## The set of feasible configurations

The set ${\mathfrak {F}} = {\mathfrak {F}}(n_{1},\dots ,n_{d}) \subseteq {\mathfrak {S}} _{\ge }$ of feasible configurations has been defined in (1.2). In this section we investigate this set. A simple observation worth to mention is that ${\mathfrak {F}}$ is closed. This follows from Proposition 1.3 and the compactness of ${\mathscr {S}}$.

### Not all configurations are feasible

When $d = 2$, we know that the singular values of a matrix and its transpose are the same, so trivially not all configurations for ${\varvec{\sigma }} _{\mathbf {X}}^{(1)}$ and ${\varvec{\sigma }}_{\mathbf {X}}^{(2)}$ are possible. The formal statement, using the introduced notation, reads, with $n_{1} \ge n_{2}$,

$$\begin{aligned} {\mathfrak {F}}(n_{1},n_{2}) = \left\{ {\varvec{\Sigma }} = ({\varvec{\sigma }} ^{(1)},{\varvec{\sigma }}^{(2)}) \in {\mathfrak {S}}_{\ge }(n_{1},n_{2}) :{\varvec{\sigma }}^{(1)} = ( {\varvec{\sigma }} ^{(2)},0,\dots ,0 ) \right\} \ne {\mathfrak {S}}_{\ge }. \end{aligned}$$

In fact, ${\mathfrak {F}}$ is an $n_{2}$-dimensional subset in the $(n_{1} + n_{2})$-dimensional set ${\mathfrak {S}}_{\ge }(n_{1},n_{2})$.

This known phenomenon in the matrix case can be used to give a qualitative proof that also for higher-order tensors not all configurations are feasible. To start, we recall a fact on the HOSVD from the literature. Let ${\mathbf {X}}$ have the left singular vector matrices $U^{(j)}_{\mathbf {X}}$ (column-wise ordered by decreasing singular values), and multilinear rank $\mathbf {r} = (r_{1},\dots ,r_{d})$. Then we can write the “economic” HOSVD as

$$\begin{aligned} {\mathbf {X}} = \left( \hat{U}^{(1)}_{\mathbf {X}}, \dots , \hat{U}^{(d)}_{\mathbf {X} }\right) \cdot \mathbf {C}, \end{aligned}$$

where $\hat{U}^{(j)}_{\mathbf {X}}$ contains only the first $r_{j}$ columns of $U^{(j)}_{\mathbf {X}}$, and the *core tensor* $\mathbf {C}$ is of size $r_{1} \times \dots \times r_{d}$. The multilinear matrix product here corresponds to the action of the tensor product operator $\hat{U}^{(1)}_{ \mathbf {X}} \otimes \dots \otimes \hat{U}^{(d)}_{\mathbf {X}}$ on ${\mathbb {R}} ^{r_{1}} \otimes \dots \otimes {\mathbb {R}}^{r_{d}}$, the explicit formulas are similar to (2.1). Note that if $\mathbf {X}$ is non-singular, $\mathbf {C}$ is just an HOSVD tensor in the orthogonally equivalent orbit of $\mathbf {X}$ as defined above. The key observation in the general case is that $\mathbf {C}$ is non-singular in ${\mathbb {R}}^{r_1 \times \dots \times r_d}$, and its higher-order singular values in every direction are given by the nonzero higher-order singular values of ${\mathbf {X}}$ [3].

Based on this fact, we can first give trivial examples of singular tensors for which the nonzero singular values in different directions are not independent of each other.

#### Lemma 3.1

Let ${\mathbf {X}}$ have multilinear rank $\mathbf {r} = (r_{1},\dots ,r_{d})$. Assume $r_{j} = 1$ for $j \ge 3$. Then $r_{1} = r_{2}$ and $\{ \sigma ^{(1)}_{1},\dots , \sigma ^{(1)}_{r_{1}} \} = \{ \sigma ^{(2)}_{1},\dots , \sigma ^{(2)}_{r_{2}} \}$.

#### Proof

Let $\mathbf {C} \in {\mathbb {R}}^{r_1 \times \dots \times r_d}$ be the economic HOSVD core tensor of ${\mathbf {X}}$. The matricizations $M_{\mathbf {C}}^{(j)}$ for $j \ge 3$ are just row vectors and have only one singular value which equals the Frobenius norm of $\mathbf {X}$. On the other hand, we have $M_{\mathbf {C}}^{(1)} = ( M_{\mathbf {C}}^{(2)} )^{\mathsf {T}}$ (up to possible permutations), which implies the result. $\square$

Since tensors with $r_{j} = 1$ for $j \ge 3$ considered in the previous lemma are naturally identified as elements of ${\mathbb {R}}^{n_{1}} \otimes { \mathbb {R}}^{n_{2}}$, that is, as matrices, the previous statement may appear rather odd at first. However, using a perturbation argument, it leads to a non-constructive proof that non-feasible configurations for higher-order singular values do exist even in the non-singular case. In fact, these configurations are of positive volume within ${\mathfrak {S}}_{\ge }$.

#### Theorem 3.2

For $n_1 \ge n_2$, consider ${\varvec{\sigma }} ^{(1)}\in {S}_{\ge }^{(1)}$ and $\varvec{\sigma }^{(2)}\in {S}_{\ge }^{(2)}$ such that

3.1 $$\begin{aligned} {\varvec{\sigma }}^{(1)} \ne ({\varvec{\sigma }}^{(2)},0,\dots ,0), \end{aligned}$$

where the number of appended zeros on the right side equals $n_1 - n_2$. Let further $\mathscr {O}_{\epsilon }^{(j)}$, $j=3,\dots ,d$, denote neighbourhoods of $\mathbf {e}_{1}^{(j)}=(1,0,\dots ,0)$ in ${S}_{\ge }^{(j)}$ of diameter at most $\epsilon >0$ (w.r.t. some norm). For $j\in \{1,2\}$, let $\mathscr {O}_{\epsilon }^{(j)}$ be similar neighbourhoods, but of ${\varvec{\sigma }}^{(1)}$ and ${\varvec{\sigma }}^{(2)}$, respectively. Then there exists $\epsilon >0$ such that

$$\begin{aligned} {\mathfrak {F}}\cap \left( \mathscr {O}_{\epsilon }^{(1)}\times \dots \times \mathscr {O} _{\epsilon }^{(d)}\right) =\emptyset , \end{aligned}$$

that is, no ${\varvec{\Sigma }}\in \mathscr {O}_{\epsilon }^{(1)}\times \dots \times \mathscr {O}_{\epsilon }^{(d)}$ is feasible.

#### Proof

Assume to the contrary that for every *n* there exists a tensor ${\mathbf {X}}_n \in {\mathscr {S}}$ such that ${\varvec{\Sigma }}_{{\mathbf {X}}_n} \in \mathscr {O}^{(1)}_{1/n} \times \dots \times \mathscr {O}^{(d)}_{1/n}$. The sequence of ${\mathbf {X}}_n$ has a convergent subsequence with a limit ${\mathbf {X}}\in {\mathscr {S}}$. By Lemma 1.3, ${\mathbf {X}}$ has higher-order singular values ${\varvec{\Sigma }}_{{\mathbf {X}}} = ({\varvec{\sigma }}^{(1)},{\varvec{\sigma }}^{(2)},\mathbf {e}^{(3)}_1,\dots ,\mathbf {e}^{(d)}_1)$. Now Lemma 3.1 applies, but is in contradiction to (3.1). $\square$

#### Remark 3.3

The condition (3.1) can hold in two cases: (i) the number of nonzero singular values in direction one and two are the same ($r_{1} = r_{2}$), but the singular values themselves are not, or (ii) $r_{1} \ne r_{2}$. The second case has some interesting implications for rectangular tensors. Assume for instance $n_{1} \ne n_{2}$. Then by Theorem 3.2 there cannot exist normalized non-singular tensors in ${\mathbb {R}}^{n_{1} \times \dots \times n_{d}}$ for which the singular value vectors ${\varvec{\sigma }}^{(j)}$ in directions $j = 3,\dots ,d$ are arbitrarily close to the corresponding unit vector $\mathbf {e}^{(j)}$. This surprising connection between mode sizes of the tensor and location of the singular value vectors is not obvious, especially given the fact that almost every tensor is non-singular (assuming (1.2)).

### A conjecture on interior points

For $d = 2$ we have seen that ${\mathfrak {F}}(n_{1},n_{2})$ is a set of measure zero within ${\mathfrak {S}}_{\ge }(n_{1},n_{2})$, even when $n_{1} = n_{2}$. One question is whether this is also true for higher-order tensors. Remarkably, the following experiment suggests that this does not need to be the case.

**Fig. 1:**
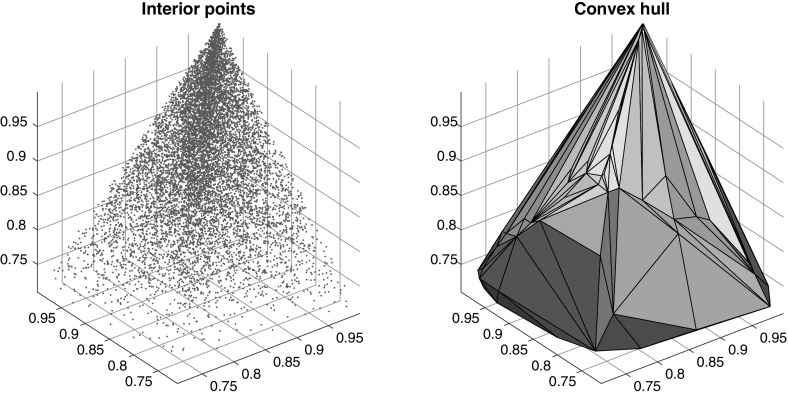
Visualizing higher-order singular values in the $2 \times 2 \times 2$ case. Plotted are the vectors $(\sigma ^{(1)}_{1},\sigma ^{(2)}_{1},\sigma ^{(3)}_{1})$ containing the largest singular values of the three directions for 10,000 random tensors ${\mathbf {X}} \in {\mathscr {S}}$. They seem to form a three-dimensional connected set. Hence, the corresponding set of $\varvec{\Sigma }_{\mathbf {X}}^{} = (\varvec{\sigma }^{(1)}_{\mathbf {X}},\varvec{\sigma }^{(1)}_{\mathbf {X}},\varvec{\sigma }^{(1)}_{\mathbf {X}})$ should be of positive volume in the three-dimensional set $\mathfrak {S}_\ge$

We generate random $2 \times 2 \times 2$ tensors ${\mathbf {X}}$ of Frobenius norm one.^2^ With probability one, the higher-order singular values ${\varvec{\Sigma }}_{\mathbf {X}} = ({\varvec{\sigma }}_{\mathbf { X}}^{(1)},{\varvec{\sigma }}_{\mathbf {X}}^{(2)},{\varvec{\sigma }}_{ \mathbf {X}}^{(3)})$ are elements of ${\mathfrak {S}}_{>}(2,2,2)$, which is a set of three dimensions and therefore can be visualized. We simply make the identification,

$$\begin{aligned} \Sigma _{\mathbf {X}} \mapsto \left( \sigma ^{(1)}_{1},\sigma ^{(2)}_{1},\sigma ^{(3)}_{1}\right) \in \left[ \tfrac{1}{ \sqrt{2}},1\right] ^{3}, \end{aligned}$$

that is, we project on the first coordinate of each singular value vector. In Fig. 1 we see these projected points for 10,000 random examples, and their convex hull computed with a Matlab integrated Delauney triangulation.

As the resulting point cloud appears three-dimensional, we suppose that the set of feasible configurations is also three-dimensional. But one can also verify in the plot that not all configurations are feasible. Above we made use of the fact that ${\varvec{\sigma }}^{(j)} = (1,0)$ (Tucker rank in the direction *j* equals one) implies ${\varvec{\sigma }}^{(i)} = {\varvec{\sigma }}^{(k)}$ for $i,k \ne j$. This can be seen in the picture as the convex polytope intersects the hyperplanes $x=1$, $y=1$ and $z=1$ in single one-dimensional facets of 45 degree.

We are led to the following conjecture.

#### Conjecture 3.4

When $d \ge 3$, and given the compatibility condition (1.2), the set ${\mathfrak {F}}(n_{1},\dots ,n_{d})$ has positive (relative) volume in ${\mathfrak {S}}_{\ge }(n_{1},\dots ,n_{d})$.

In fact, the following seems likely (under the same assumptions).

#### Conjecture 3.5

For generic ${\mathbf {X}} \in {{\mathscr {S}}}$, ${\varvec{\Sigma }}_{\mathbf {X}}$ is a (relative) interior point of ${\mathfrak {F}}(n_{1},\dots ,n_{d})$ within ${\mathfrak {S}}_{\ge }(n_{1},\dots ,n_{d})$.

#### Remark 3.6

During revision of the paper, a possible strategy to prove this conjecture has been revealed. It is based on the observation that $\varvec{\Sigma }_{{\mathbf {X}}}$ is a relative interior point of $\mathfrak {F}(n_{1},\dots ,n_{d})$ if and only if the map $g({\mathbf {X}}) = ( G_{{\mathbf {X}}}^{(1)} ,\dots , G_{{\mathbf {X}}}^{(d)} )$ (that has already been considered in (2.4)) is locally surjective when regarded as a map from the unit sphere ${\mathscr {S}}$ to the Cartesian product of hyperplanes $\{A^{(j)} \in {\mathbb {R}}^{n_j \times n_j}_{\text {sym}} :{{\mathrm{{tr}}}}(A^{(j)}) = 1\}$. In other words, one has to show that the rank of the derivative $g'({\mathbf {X}})$, when restricted to the tangent space $T_{{\mathbf {X}}} {\mathscr {S}}$, equals the maximum possible value $\alpha = \left( \sum _{j=1}^d \frac{1}{2} n_j(n_j + 1) \right) - d$. A sufficient condition for this is that $g'({\mathbf {X}})$ is of rank $\alpha + 1$ on ${\mathbb {R}}^{n_1 \times \dots \times n_d}$. However, as $g'({\mathbf {X}})$ depends polynomially on the entries of ${\mathbf {X}}$, the function ${\mathbf {X}}\mapsto {{\mathrm{{rank}}}}(g'({\mathbf {X}}))$ achieves its maximum value for almost all ${\mathbf {X}}$. Since it is bounded by $\alpha + 1$, it is therefore enough to find a single tensor ${\mathbf {X}}$ for which rank $\alpha + 1$ is achieved. In this way, one can validate Conjecture 3.5 for different configurations of $n_1,\dots ,n_d$ by constructing random ${\mathbf {X}}$ and evaluating the rank of $g'({\mathbf {X}})$ numerically. A rigorous proof would have to confirm this numerical rank for “simple” candidates ${\mathbf {X}}$, which we were able to do for $2 \times 2 \times 2$ tensors so far. This approach shall be subject of a future work.

### Alternating projection method

Even in the case that one would be given the information that a configuration ${\varvec{\Sigma }}=({\varvec{\sigma }}^{(1)},\dots ,{ \varvec{\sigma }}^{(d)})\in {\mathfrak {S}}_{\ge }$ is feasible, the question remains how to construct a corresponding tensor. Note that the suggested strategy to prove Conjecture 3.5 by showing full rank of (2.4) may not provide an explicit way for perturbing singular values in single directions.

A (currently) heuristic approach can be taken via the method of alternating projections. It is based on an alternative viewpoint on Problem 1.4: Given ${\varvec{\sigma }}^{(j)} \in {S}^{(j)}_{\ge }$ for $j=1,\dots ,d$, the configuration ${\varvec{\Sigma }} = ({\varvec{\sigma }}^{(1)},\dots ,{\varvec{\sigma }}^{(d)})$ is feasible, if and only if there exists a tensor ${\mathbf {X}}$ such that

3.2 $$\begin{aligned} {\mathbf {X}} \in \bigcap _{j=1}^{d} {\mathscr {M}}_{{\varvec{\sigma }} ^{(j)}}^{(j)}, \end{aligned}$$

where ${\mathscr {M}}_{\varvec{\sigma }}^{(j)}$ denotes the set of all tensors with mode-*j* singular values ${\varvec{\sigma }}$. More concretely,

$$\begin{aligned} {\mathscr {M}}_{\varvec{\sigma }}^{(j)} =\left\{ {\mathbf {X}} :M_{{ \mathbf {X}}}^{(j)} = U {{\mathrm{{diag}}}}({\varvec{\sigma }}) V^{\mathsf {T}}, \ U \in { \mathrm {O}}(n_{j}), \ {V^{\mathsf {T}}V = I_{n_j}} \right\} . \end{aligned}$$

The method of alternating projections tries to find ${\mathbf {X}}$ satisfying (3.2) by successively projecting on the sets ${ \mathscr {M}}_{{\varvec{\sigma }}^{(j)}}^{(j)}$. It hence takes the form

3.3 $$\begin{aligned} {\mathbf {X}}_{k+1} = \left( \Pi _{{\varvec{\sigma }}^{(d)}}^{(d)} \circ \dots \circ \Pi _{{\varvec{\sigma }}^{(1)}}^{(1)}\right) ({\mathbf {X}} _{k}), \end{aligned}$$

where $\Pi _{{\varvec{\sigma }}}^{(j)} :{\mathbb {R}}^{n_{1} \times \dots \times n_{d}} \rightarrow {\mathscr {M}}_{\varvec{\sigma }}^{(j)}$ is a metric projection on the set ${\mathscr {M}}_{{\varvec{\sigma }}}^{(j)}$, that is, $\Pi _{{\varvec{\sigma }}}^{(j)}({\mathbf {Y}})$ returns a best approximation of ${\mathbf {Y}}$ in the set ${\mathscr {M}}_{\varvec{ \sigma }}^{(j)}$. A best approximation in Frobenius norm can be obtained by simply replacing the singular values of $M_{{\mathbf {Y}}}^{(j)}$ with ${ \varvec{\sigma }}$:

3.4 $$\begin{aligned} M_{\Pi _{\varvec{\sigma }}^{(j)}({\mathbf {Y}})}^{(j)} = U_{\mathbf {Y} }^{(j)} {{\mathrm{{diag}}}}\left( {\varvec{\sigma }}\right) (V_{\mathbf {Y}}^{(j)})^{\mathsf {T}}. \end{aligned}$$

Moreover, if $\sigma _{\mathbf {Y}}^{(j)} \in {S}_{>}^{(j)}$, this best approximation is unique. To prove these assertions, note that the best approximation problem in Frobenius norm is equivalent to maximizing the trace of $U {{\mathrm{{diag}}}}({\varvec{\sigma }}) V^{\mathsf {T}} ( M_{{\mathbf {Y}}}^{(j)} )^{ \mathsf {T}}$ over all $U \in {\mathrm {O}}(n_{j})$ and $V \in \mathbb {R}^{n_j^c}$, $V^{\mathsf {T}}V = I_{n_j}$. The von Neumann trace inequality  [15, 23] states that the upper bound for this quantity is ${\varvec{\sigma }}^{\mathsf {T}} {\varvec{\sigma }}_{\mathbf {Y}}^{(j)}$ . Moreover, equality is achieved at *U*, *V* if and only if $M_{{\mathbf {Y}} }^{(j)} = U {{\mathrm{{diag}}}}({\varvec{\sigma }}_{\mathbf {Y}}^{(j)}) V^{\mathsf {T}}$, see [4, Remark 1.2]. Hence $U = U^{(j)}_{\mathbf {Y}}$ and $V = V^{(j)}_{\mathbf {Y}}$ are unique in the case that ${\varvec{\sigma }}^{(j)}_{ \mathbf {Y}} \in {S}^{(j)}_{>}$.

Although the interpretation as an alternating projection method is nice, we remark that the multiplication by $U_{\mathbf {Y}}^{(j)}$ in  (3.4) could be omitted in practice. It is an easy induction to show that in this case an orthogonally equivalent sequence of tensors would be produced.

Even assuming that intersection points exist, we are currently not able to provide local or global convergence results for the alternating projection method  (3.3). Instead, we confine ourselves with three numerical illustrations.

*Recovering a feasible configuration*

**Fig. 2:**
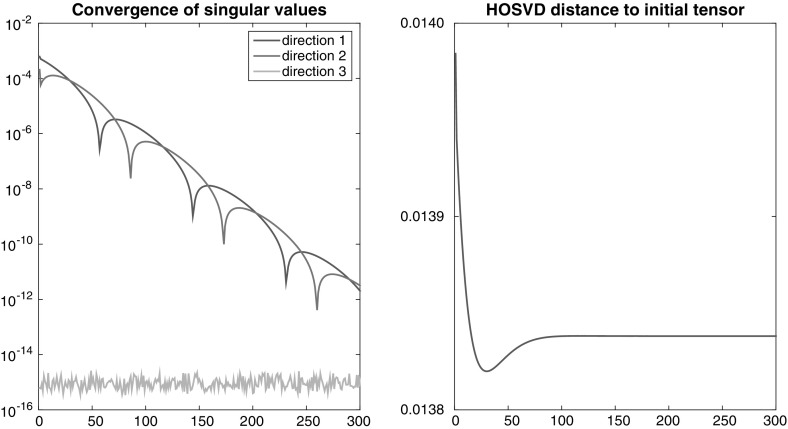
Recovering a feasible configuration in ${\mathbb {R}}^{30 \times 30 \times 30}$ via alternating projection. *Left* errors of singular value vectors. *Right* maximum difference between absolute values of entries of HOSVD core tensors of iterates and the generating tensor that provided the feasible configuration. As it does not go to zero, the limiting tensor is not orthogonally equivalent to the generating tensor

To obtain a feasible configuration ${\varvec{\Sigma }}$, we create a norm-one tensor ${\mathbf {X}}$ and take its higher-order singular values, ${ \varvec{\Sigma }}={\varvec{\Sigma }}_{\mathbf {X}}$. Then we run the iteration (3.3) starting from a random initialization, and measure the errors $\Vert {\varvec{\sigma }}_{{ \mathbf {X}}_{k+1}}^{(j)}-{\varvec{\sigma }}_{\mathbf {X}}^{(j)}\Vert _{2}$ (Euclidean norm) after every full cycle of projections. Since $\Pi _{{ \varvec{\sigma }}^{(3)}}^{(3)}$ is applied last, the singular values in direction three are always correct at the time the error is measured. The question is whether also the singular values in the other directions converge to the desired target values. The left plot in Fig. 2 shows one typical example of error curves observed in this kind of experiment in ${ \mathbb {R}}^{30\times 30\times 30}$. We see that the sequence ${\varvec{ \Sigma }}_{{\mathbf {X}}_{k}}$ converges to ${\varvec{\Sigma }}$, hence every cluster point of the sequence ${\mathbf {X}}_{k}$ will have the desired higher-order singular values. So far, we have no theoretical explanation for the shifted peaks occurring in the curves.

Since our initial guess is random, we do not expect that the generating ${ \mathbf {X}}$ or an orthogonally equivalent tensor will be recovered. To verify this, we make use of Proposition 2.6 and measure the error $\max _{i_{1},\dots ,i_{d}} |\vert \hat{X} _{i_{1},\dots ,i_{d}} \vert - \vert (\hat{X}_{k+1})_{i_{1},\dots ,i_{d}} \vert |$ after every loop, where $\hat{{\mathbf {X}}} _{k+1}$ and $\hat{{\mathbf {X}}}$ are HOSVD representatives in the corresponding orbits of orthogonal equivalence. The right plot in Fig.  shows this error curve, and we can see it does not tend to zero. By Proposition 2.6, the limiting tensor is hence not orthogonally equivalent to $\mathbf {X }$. Since this behaviour was observed being typical, the alternating projection method can be suggested as a practical procedure to construct tensors having the same higher-order singular values without being orthogonally equivalent.

Experiments with tensors of order $d=4$ and larger lead to similar results, but they quickly become computationally expensive as SVDs of large matrices have to be calculated.

**Fig. 3:**
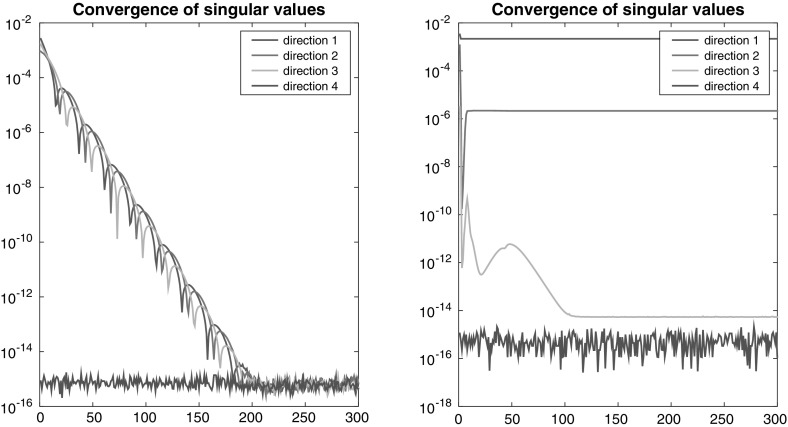
Experiments in ${\mathbb {R}}^{10\times 10\times 10\times 10}$. *Left* perturbation of a given feasible configuration $\varvec{\Sigma }$ by $\mathscr {O}(\epsilon )$ with $\epsilon = 10^{-3}$. *Right* infeasible configuration $\sigma ^{(j)} = (1,0,\dots ,0) + \mathscr {O}(\epsilon _j)$ obtained using $\epsilon _3=\epsilon _4 = 10^{-6}$, but $\epsilon _1 = \epsilon _2 = 10^{-3}$

*Perturbation of a feasible configuration*

To support Conjecture 3.5, we now consider random perturbations

$$\begin{aligned} {\varvec{\Sigma }}_{\epsilon }=\left( {\varvec{\sigma }} _{\epsilon }^{(1)},\dots ,{\varvec{\sigma }}_{\epsilon }^{(d)}\right) ={\varvec{ \Sigma }}+\mathscr {O}(\epsilon )\in {\mathscr {S}}_{\ge } \end{aligned}$$

of a known feasible configuration ${\varvec{\Sigma }}\in {\mathfrak {F}}\cap {\mathfrak {S}}_{>}$ (obtained again from a random tensor ${\mathbf {X}}\in {\mathscr {S}}^{*}$).^3^ According to the conjecture, we expect that for small $\epsilon$ the configuration ${\varvec{\Sigma }}_{\epsilon }$ is also feasible, so a corresponding tensor may be found by the alternating projection method  (3.3). This can be verified in numerical experiments. The left plot in Fig. 3 shows the errors $\Vert {\varvec{\sigma }}_{{\mathbf {X }}_{k+1}}^{(j)}-{\varvec{\sigma }}_{\epsilon }^{(j)}\Vert _{F}$ for one experiment in ${\mathbb {R}}^{10\times 10\times 10\times 10}$ using $\epsilon = 10^{-3}$.

*Infeasible configuration*

When conducting our experiments with the alternating projection method, we made the experience that with high probability even a randomly generated configuration will be feasible. Indeed, Fig. 1 supports this in the $2 \times 2 \times 2$ case, as the feasible configurations seem to make up a rather large fraction in ${\mathfrak {S}}_{\ge }(2,2,2)$.

To construct an infeasible configuration we therefore mimic the proof of Theorem 3.2: we generate ${\varvec{ \sigma }}^{(j)}$ as $(1,0,\dots ,0)+\mathscr {O}(\epsilon _{j})$ (as described in Footnote 3), where we use very small $\epsilon _{j}$ for $j\ge 3$, e.g., $\epsilon _j = 10^{-6}$. By the arguments presented above this should also enforce ${\varvec{\sigma }}^{(1)}$ to be close to ${\varvec{\sigma }}^{(2)}$ to ensure feasibility. To impede this, we use larger $\epsilon _1$ and $\epsilon _2$ instead, e.g., $\epsilon _{1}=\epsilon _{2}=10^{-3}$ (an alternative would be to generate ${ \varvec{\sigma }}^{(1)}$ and ${\varvec{\sigma }}^{(2)}$ completely random). Our results suggest that this indeed results in an infeasible configuration. Accordingly, the alternating projection method fails. The right plot in Fig. 3 shows the outcome of one experiment, again in $\mathbb {R}^{10 \times 10 \times 10 \times 10}$.

## Footnotes

1 For $d=2$, i.e., matrices, it is the case: as  (1.2) is assumed, we have $n_{1}=n_{2}=n$, and two square matrices have the same singular values if and only if they are orthogonally equivalent. The formula gives $n^{2}-2n+1$ which, however, only equals $n^{2}-n-(n-1)=\dim ({\mathrm {O}}(n\times n))-(n-1)$. The reason is that in the matrix case we know that the singular values of ${\mathbf {X}}$ and ${ \mathbf {X}}^{\mathsf {T}}$ are the same. Hence the feasible set is only of dimension $n-1$, and not of dimension $2(n-1)$ (the argument will be repeated in Sect. 3.1). For tensors, however, we conjecture that the dimension of ${\mathfrak {F}}$ is indeed $(n_{1}+\dots +n_{d})-d$, see Sect. 3.2.

2 For our experiments we made use of the Tensor Toolbox [2] in Matlab.

3 Practically, ${\varvec{\Sigma }}_\epsilon$ was generated by normalizing and sorting the perturbed $\left|\sigma ^{(j)} + \mathscr {O}(\epsilon )\right|$.

## References

[1] Bachmayr M, Dahmen W (2015). Adaptive near-optimal rank tensor approximation for high-dimensional operator equations. Found. Comput. Math..

[2] Bader, B.W., Kolda, T.G., et al.: Matlab tensor toolbox version 2.6 (2015). http://www.sandia.gov/~tgkolda/TensorToolbox/

[3] De Lathauwer, L., De Moor, B., Vandewalle, J.: A multilinear singular value decomposition. SIAM J. Matrix Anal. Appl. **21**(4), 1253–1278 (2000, electronic)

[4] de Sá EM (1994). Exposed faces and duality for symmetric and unitarily invariant norms. Linear Algebra Appl..

[5] de Silva V, Lim L-H (2008). Tensor rank and the ill-posedness of the best low-rank approximation problem. SIAM J. Matrix Anal. Appl..

[6] Dieudonné J (1972). Treatise on Analysis.

[7] Grasedyck, L.: Hierarchical singular value decomposition of tensors. SIAM J. Matrix Anal. Appl. **31**(4), 2029–2054 (2009/2010)

[8] Hackbusch W (2012). Tensor Spaces and Numerical Tensor Calculus.

[9] Hackbusch W, Kühn S (2009). A new scheme for the tensor representation. J. Fourier Anal. Appl..

[10] Hitchcock FL (1927). The expression of a tensor or a polyadic as a sum of products. J. Math. Phys..

[11] Hitchcock FL (1927). Multiple invariants and generalized rank of a $$p$$-way matrix or tensor. J. Math. Phys..

[12] Koch O, Lubich C (2010). Dynamical tensor approximation. SIAM J. Matrix Anal. Appl..

[13] Kolda TG, Bader BW (2009). Tensor decompositions and applications. SIAM Rev..

[14] Lubich C, Rohwedder T, Schneider R, Vandereycken B (2013). Dynamical approximation by hierarchical Tucker and tensor-train tensors. SIAM J. Matrix Anal. Appl..

[15] Mirsky L (1975). A trace inequality of John von Neumann. Monatsh. Math..

[16] Oseledets I, Tyrtyshnikov E (2010). TT-cross approximation for multidimensional arrays. Linear Algebra Appl..

[17] Oseledets IV (2011). Tensor-train decomposition. SIAM J. Sci. Comput..

[18] Oseledets IV, Tyrtyshnikov EE (2009). Breaking the curse of dimensionality, or how to use SVD in many dimensions. SIAM J. Sci. Comput..

[19] Oseledets, I.V., Tyrtyshnikov, E.E.: Recursive decomposition of multidimensional tensors. Dokl. Akad. Nauk **427**(1), 14–16 (2009, in Russian) [English translation in: Dokl. Math. **80**(1), 460–462 (2009)]

[20] Rohwedder T, Uschmajew A (2013). On local convergence of alternating schemes for optimization of convex problems in the tensor train format. SIAM J. Numer. Anal..

[21] Schneider R, Uschmajew A (2014). Approximation rates for the hierarchical tensor format in periodic Sobolev spaces. J. Complex..

[22] Tucker LR (1966). Some mathematical notes on three-mode factor analysis. Psychometrika.

[23] von Neumann J (1937). Some matrix-inequalities and metrization of matrix-space. Tomsk. Univ. Rev..

